# Response to: “timing of administration of indocyanine green for fluorescence-guided surgery in pancreatic cancer: response to Shirakawa et al.”

**DOI:** 10.1186/s12893-020-00815-7

**Published:** 2020-07-14

**Authors:** Sachiyo Shirakawa, Hirochika Toyama, Masahiro Kido, Takumi Fukumoto

**Affiliations:** Division of Hepato-Biliary-Pancreatic Surgery, Department of Surgery, Kobe University Graduate School of Medicine, 7-5-2 Kusunoki-cho Chuo-ku, Kobe Hyogo, Japan

## Abstract

This is the response article to correspondence article received for our published article in BMC surgery titled “A prospective single-center protocol for using near-infrared fluorescence imaging with indocyanine green during staging laparoscopy to detect small metastasis from pancreatic cancer”. Peter L. Labib, MBChB pointed out the necessity to administer indocyanine green intravenously in separate timing for detection of metastasis in liver and peritoneum. Preoperative injection is suitable to detect hepatic metastasis and intraoperative injection is reported to be well suited to detect peritoneal metastasis. However, we could not find the usefulness of intraoperative injection of indocyanine green for detecting peritoneal metastasis in cases with staging laparoscopy prior to this study. We employed this study protocol with only preoperative injection of indocyanine green to simplify the procedure with consideration of probably more frequent cases of hepatic metastasis that is difficult to detect with white-light imaging than those of peritoneal metastasis.

Dear Editor,

We would like to thank Peter L. Labib, MBChB, for addressing my recent article about near-infrared (NIR) fluorescence imaging with indocyanine green (ICG) during staging laparoscopy for pancreatic ductal adenocarcinoma [1]. We also thank the author for describing details of NIR imaging using ICG for each site of the lesion, liver, peritoneum, and pancreas, regarding the timing of ICG injection.

I agree with the author about the timing of ICG injection for detecting peritoneal metastases. We performed staging laparoscopy in three cases with peritoneal metastases using two ICG injection steps prior to this prospective study. We found apparent peritoneal metastases in these cases with white-light imaging during the staging laparoscopy, and there was no fluorescent peritoneal lesion through NIR imaging with preoperative ICG injection on the previous day. Then, we added intraoperative ICG injections of 0.25 mg/kg of patient weight after thorough exploration for hepatic metastasis with white-light and NIR imaging to check the fluorescence of peritoneal metastasis. In these cases, we could find no lesion positive for ICG fluorescence on the peritoneum. As the author described, Liberale et al. found 21 of 24 malignant solid peritoneal nodules to be positive for fluorescence with intraoperative ICG injection in colorectal cancer, and the surgery was modified based on NIR imaging in 4 of 14 patients [2]. Veys et al. reported that NIR imaging with intraoperative ICG injection could detect peritoneal metastases from ovarian cancer [3]. Based on previous reports, NIR imaging with intraoperative ICG injection is thought to be useful for detecting peritoneal metastasis of adenocarcinoma. However, we employed this study protocol with only preoperative ICG injection and without intraoperative injection, to simplify the procedure. We expected more frequent cases of hepatic metastasis that is difficult to detect with white-light imaging than those of peritoneal metastasis. Our study mainly aimed to detect hepatic metastasis.

We will consider the author’s proposal to add intraoperative ICG injection to our protocol, depending on the frequency of occult peritoneal metastasis. A total of 20 patients were enrolled in our present study until now and no patient developed new peritoneal metastasis within at least three months after the staging laparoscopy. We understand that visually occult peritoneal metastasis during the staging laparoscopy was unusual in our cohort. Hence, we did not change our protocol up to this time. We thank the authors for their interest and productive comments.

## Data Availability

Not applicable.

## Abbreviations

NIR: Near infrared
ICG: Indocyanine green
